# Recurrent respiratory tract infections in children might be associated with vitamin A status: a case-control study

**DOI:** 10.3389/fped.2023.1165037

**Published:** 2024-01-05

**Authors:** Xuguang Zhang, Xuezheng Dai, Xianan Li, Xun Xie, Yiru Chen, Yanping Chen, Haoyang Guan, Yan Zhao

**Affiliations:** ^1^ Department of Child Healthcare, The Sixth Affiliated Hospital of Harbin Medical University, Harbin, Heilongjiang, China; ^2^ Department of Nutrition and Food Hygiene, School of Public Health, Harbin Medical University, Harbin, Heilongjiang, China

**Keywords:** vitamin A insufficiency, vitamin A deficiency, recurrent respiratory tract infections, children, dietary intake

## Abstract

**Background:**

Recurrent respiratory tract infections (RRTIs) are common in children and its development might be associated with vitamin A deficiency according to recent research. The aim of this study was to understand the relation between vitamin A status and RRTIs in children, and the relation between dietary intake of vitamin A and RRTIs.

**Methods:**

2,592 children aged 0.5–14 years from Heilongjiang province of China participated in the survey. The RRTI group consisted of 1,039 children with RRTIs, while 1,553 healthy children were included in the control group. The levels of serum vitamin A were determined by high performance liquid chromatography (HPLC); dietary information was collected with the Food Frequency Questionnaire (FFQ).

**Results:**

Serum vitamin A concentration in the RRTI group was significantly lower than that in the control group (0.27 ± 0.09 mg/L vs. 0.29 ± 0.09 mg/L) (*P *< 0.01). The levels of vitamin A was obviously associated with the occurrence of RRTIs. The odds ratios (ORs) for vitamin A insufficiency and deficiency were 1.32 (95% CI: 1.09–1.60) and 1.95 (95% CI: 1.50–2.55) respectively; whereas 1.48 (95% CI: 1.13–1.94) and 6.51 (95% CI: 4.18–10.14) respectively, in children with current respiratory tract infection (RTI) symptoms. Even an insufficient intake of animal liver was associated with lower RRTIs [OR: 0.45 (95% CI: 0.38–0.53)], while only an excessive intake of meat had the same effect [OR: 0.85 (95% CI: 0.68–1.06)].

**Conclusions:**

Low serum vitamin A concentration was associated with high incidence of RRTIs in children in northeast China; low serum vitamin A concentrations and the current RTI symptoms were associated with the development of RRTIs; and low intakes of vitamin A-rich foods were also associated with the development of RRTIs.

## 1 Introduction

Recurrent respiratory tract infections (RRTIs) are common in children (1, 2) and defined as any upper or lower respiratory tract infections (RTIs) that frequently occur within a period of a year, a frequency far beyond the normal range (3–5). In recent years, the incidence of RRTIs has been increasing, and it is shown that the rates of RRTIs among children in northeast China ranged from 17.8% to 18.7% (6). The pathogenesis of RRTIs is complex, involving anatomy and physiology of the respiratory system, vitamin or trace element deficiency, genetic and environmental factors, and impaired immunity (2, 7). The deficiencies of immunoglobulin G (IgG) subclasses, especially IgG2 and specific polysaccharide antibody are common in children with RRTIs. Children with the deficiency in secretory IgA alone and/or IgG subclass are prone to have RRTIs (8, 9). Asthma and allergic diseases are important risk factors for RRTIs (10). A number of underlying conditions can also lead to RRTIs, e.g., primary or secondary immunodeficiency, reflux and aspiration primary ciliary dyskinesia, bronchopulmonary dysplasia, and congenital cardiac defects. It is noteworthy that RRTIs in most children result from immature immune systems at a developmental stage without underlying diseases (11).

Vitamin A (VA) is an essential micronutrient that plays an important role in a wide array of physiological processes, including vision, immune response, cell differentiation and proliferation, intercellular communication, and reproduction (12). Vitamin A deficiency (VAD) increases the risk of infectious diseases in children and in the long term, their mortality rates (13). Currently, VAD remains a major public health threat (14). WHO recommends that infants aged 6–11 months should be supplemented with 100,000 IU (30 mg RE) vitamin A every 6 months, and children aged 12–59 months should be supplemented with 200,000 IU (60 mg RE) vitamin A every 4–6 months to reduce child morbidity and mortality (15). Serum VA deficiency is closely related to immune impairment (16). It damages the humoral and cell-mediated immune system through direct and indirect effects on phagocyte and T cells, thus increasing the possibility of RRTI due to injury (17).

Most previous studies have focused on determining the relation between VA concentrations and RTIs (18, 19), whereas only a few studies on the relation between serum VA levels and RRTIs (20–22). In addition, the association between threshold dietary intake of vitamin A and respiratory outcomes is unclear. In this study, we explored the relation between serum VA levels and dietary intake in children and RRTIs, providing strong evidence for appropriate VA supplementation for the prevention of RRTIs.

## 2 Methods

### 2.1 Subjects and clinical assessment

A total of 2,592 children aged 0.5–14 years were recruited from the outpatient department at Harbin Children's Hospital (Heilongjiang, China) via a face-to-face questionnaire answered by their parents or guardians. The period of enrollment ranged from Feb 2015 to Jan 2018.

Assessment was based on the diagnostic criteria for RRTIs according to the consensus guidelines for the treatment of RRTIs in children in China (23). A child was diagnosed as recurrent upper respiratory tract infection if meeting at least one of the following conditions: seven or more episodes of upper respiratory tract infections per year in children aged 0–2 years, six or more in those aged 3–5 years, five or more in those aged 5–14 years. Recurrent lower respiratory tract infection, including tracheobronchitis and pneumonia, was defined as two or more lower respiratory tract infection episodes per year. The interval between two infections should be longer than 7 days. Children with underlying diseases, including inhalation allergy or allergic asthma, primary immunodeficiency, chronic respiratory diseases, other chronic conditions, or those who had recently been administered with gamma globulin, blood products, hormones, or using therapeutic dose of vitamin A followed by a serum vitamin A test, were excluded.

Of the 2,592 children, 1,039 had RRTIs and the other 1,553 without RRTIs consisted of the control group. Height, weight and levels of VA, were investigated in the survey. The presence of respiratory infection symptoms (e.g., fever, coughing, sneezing, nasal congestion, runny nose, pharyngeal congestion, etc.) was recorded at the time of the child's visit.

### 2.2 Calculation of BMI *z*-scores

Height and weight of the participants were documented and body mass index (BMI) was calculated as body weight (kg)/height squared (m^2^).

BMI was also assessed using the *z*-score based on WHO standards for all the participants. For children under 5 years old: overweight is weight-for-height greater than 2 standard deviations (SD) above WHO Child Growth Standards median; and obesity is weight-for-height greater than 3 SD above WHO Child Growth Standards median. For children between 5 and 14 years old: overweight is weight-for-height greater than 1 SD above WHO Child Growth Standards median; and obesity is weight-for-height greater than 2 SD above WHO Child Growth Standards median.

### 2.3 Determination of serum vitamin A

Blood samples (3 ml per child) were taken via antecubital venipuncture, stored cold in cool box for less than 2 h. After centrifuged at room temperature for 10 min at 1,200 × g, the upper serum was pipetted and stored at 4°C for less 24 h. Samples were kept at room temperature for 30 min before detection. Retinol was extracted from serum with hexane, followed by deproteinization, and evaporated to dryness with nitrogen gas. The residue was dissolved in 0.2 ml ethanol. Retinyl acetate dissolved in ethanol was used as the internal standard (Thermo Fisher Scientific, USA). Serum levels of retinol were determined by HPLC (LC-20AD; Shimadzu, Japan) with C8 column (Agilent, 1.7 µm, 2.1 × 50 mm) at 25°C and with a ultraviolet detector at wavelength of 325 nm in Heilongjiang Harmony Health Medical Laboratory. The elution was carried out in the solution of 95% methanol and 5% water at a flow rate of 1 ml/min. The sensitivity of detection was assessed with the inter-batch coefficient of variation (CV) of 3.7% and between batches CV of 5.1%. The retinol (Sigma, USA) was used as standard for determination.

### 2.4 Diagnostic criteria of serum vitamin A status

Based on the WHO diagnosis criteria of VA insufficiency, the serum level of vitamin A at more than 0.3 mg/L, 0.2–0.3 mg/L and less than 0.2 mg/L was considered as normal, insufficient and deficient, respectively.

### 2.5 Dietary intake information

Intake of main staples, milk, meat, eggs, vegetables and fruits was investigated with a food frequency questionnaire (FFQ). The intake was assigned according to the recommended daily intakes for children of different ages by the Chinese Nutrition Society (24), combined with the frequency of food intake. The daily intakes within the range of recommendation were considered adequate; otherwise, it would be insufficient. Daily intakes more than the recommendation were considered excessive. None intakes of milk, egg and animal liver were recorded.

### 2.6 Statistical analysis

SPSS 24.0 software was used for all statistical analysis of the data. Quantitative data were expressed as mean ± SD or median (inter-quartile range, IQR) and those that followed a normal distribution were analyzed with the *t*-test; otherwise, the Mann–Whitney *U* test was employed. The chi-squared test was used to compare differences in the distribution of qualitative variables between groups. Values with *P *< 0.05 were considered significant for all statistical tests. Logistic regression was used to determine odds ratios (OR) and 95% confidence intervals (CI) to evaluate the association of RRTIs with the serum levels of VA, as well as the association between food intake and RRTIs.

Factor analysis (principal component analysis, PCA) was applied to explore the dietary pattern for the study population. The analysis was based on the daily intake of 17 VA-rich foods in 5 categories. The eigenvalues and the scree plot were used to decide on the number of factors retained. Varimax rotation was performed and the adequacy of the data was determined with the Kaiser-Meyer-Olkin (KMO) index and Bartlett's sphericity test. Factor loadings <0.60 were excluded. Dietary patterns were named according to the characteristics of the included food groups. Dietary patterns were further categorized into quartiles, where quartile 1 represented low intake and quartile 4 high adherence to the dietary pattern. Quartiles of adherence allow for better evaluation of dietary intake.

## 3 Results

### 3.1 Demographic characteristics

Demographic and clinical characteristics of the children in the study were summarized in Table 1. 2,592 children were included and divided into the control group (*n* = 1,553) and RRTI group (*n* = 1,039), with serum VA levels of 0.29 ± 0.09 mg/L and 0.27 ± 0.09 mg/L (*P *< 0.01), respectively. The occurrence of RRTIs was observed more frequently in the age of 3–5 years (38.1% vs. 28.4% in the control group). The percentage of children with fever, current RTI symptoms or vitamin A deficiency was higher in RRTI group than in control group, while the percentage with VA supplementation was lower in the children with RRTIs (*P *< 0.05). Sex, urban/rural locality and BMI *z*-scores were not statistically different.

**Table 1:** Demographic and clinical characteristics of the children in this study.

Characteristics	Control group (*n* = 1,553)	RRTI group (*n* = 1,039)	*P*
Age, year^a^	4.42 (1.92, 7.91)	4.92 (3.00, 7.30)	0.002
0–2	538 (34.6)	256 (24.6)	0.00
3–5	441 (28.4)	396 (38.1)	
6–14	574 (37.0)	387 (37.3)	
Sex			0.22
Boy	933 (60.1)	649 (62.5)	
Girl	620 (39.9)	390 (37.5)	
Residency			0.82
Urban	1,270 (81.8)	846 (81.4)	
Rural	283 (18.2)	193 (18.6)	
BMI *z*-scores			0.77
Normal	1,303 (83.9)	865 (83.3)	
Overweight	176 (11.3)	127 (12.2)	
Obesity	74 (4.8)	47 (4.5)	
Fever			0.00
Yes	167 (10.8)	356 (34.3)	
No	1,386 (89.2)	683 (65.7)	
Vitamin A (mg/L)^b^	0.29 ± 0.09	0.27 ± 0.09	0.00
Sufficiency	725 (46.7)	351 (33.8)	0.00
Insufficiency	639 (41.1)	478 (46.0)	
Deficiency	189 (12.2)	210 (20.2)	
Current symptoms of respiratory infection			0.00
Yes	575 (37.0)	744 (71.6)	
No	978 (63.0)	295 (28.4)	
Vitamin A preparation (%)			0.001
Yes	367 (23.6)	189 (18.2)	
No	1.186 (76.4)	850 (81.8)	

Data are expressed as *n* (%).

^a^ Median (Q1, Q3).

^b^ Mean ± SD.

Age, sex, urban/rural locality and BMI *z*-scores in the RRTI group compared to controls were not statistically different (*P *> 0.05) in children with adequate (*n* = 1,076) and deficient (*n* = 399) serum VA levels. Among children with serum VA insufficiency (*n* = 1,117), the age median of the control and RRTI groups was 4.17 (1.58, 7.25) and 5.17 (3.08, 7.69) years old, respectively. In the children with RRTIs, the percentage of fever or current RTI symptoms was higher in the condition of vitamin A insufficiency and deficiency than sufficiency (36.0% and 40.0% vs. 28.5%). On the contrary, the percentage of VA supplementation was the lowest with vitamin A deficiency (4.8%) (Table 2).

**Table 2:** Comparison of the basic characteristics of children with different serum vitamin A levels.

Characteristics	Vitamin A sufficiency		*P*	Vitamin A insufficiency		*P*	Vitamin A deficiency		*P*
	Controls (*n* = 725)	RRTI (*n* = 351)		Controls (*n* = 639)	RRTI (*n* = 478)		Control (*n* = 189)	RRTI (*n* = 210)	
Age, year^a^	5.10 (2.14, 8.90)	5.10 (3.08, 8.10)	0.48	4.17 (1.58, 7.25)	5.17 (3.08, 7.69)	0.00	3.83 (1.67, 5.75)	4.13 (2.25, 6.12)	0.23
0–2	227 (31.3)	84 (23.9)	0.00	239 (37.4)	107 (22.4)	0.00	72 (38.1)	65 (31.0)	0.25
3–5	182 (25.1)	128 (36.5)		183 (28.6)	180 (37.6)		76 (40.2)	88 (41.9)	
6–14	316 (43.6)	139 (39.6)		217 (34.0)	191 (40.0)		41 (21.7)	57 (27.1)	
Sex			0.75			0.33			0.44
Boy	445 (61.4)	219 (62.4)		369 (57.7)	290 (60.7)		119 (63.0)	140 (66.7)	
Girl	280 (38.6)	132 (37.6)		270 (42.3)	188 (39.3)		70 (37.0)	70 (33.3)	
Residency			0.29			0.20			0.21
Urban	575 (79.3)	288 (82.1)		526 (82.3)	379 (79.3)		169 (89.4)	179 (85.2)	
Rural	150 (20.7)	63 (17.9)		113 (17.7)	99 (20.7)		20 (10.6)	31 (14.8)	
BMI *z*-scores			0.49			0.14			0.53
Normal	582 (80.3)	288 (82.1)		556 (87.0)	396 (82.8)		165 (87.3)	181 (86.2)	
Overweight	92 (12.7)	45 (12.8)		63 (9.9)	60 (12.6)		21 (11.1)	22 (10.5)	
Obesity	51 (7.0)	18 (5.1)		20 (3.1)	22 (4.6)		3 (1.6)	7 (3.3)	
Fever			0.00			0.00			0.17
Yes	44 (6.1)	100 (28.5)		60 (9.4)	172 (36.0)		63 (33.3)	84 (40.0)	
No	681 (93.9)	251 (71.5)		579 (90.6)	306 (64.0)		126 (66.7)	126 (60.0)	
Current symptoms of respiratory infection			0.00			0.00			0.00
Yes	253 (34.9)	232 (66.1)		284 (44.4)	347 (72.6)		38 (20.1)	165 (78.6)	
No	472 (65.1)	119 (33.9)		355 (55.6)	131 (27.4)		151 (79.9)	45 (21.4)	
Vitamin A supplementation			0.001			0.04			0.00
Yes	129 (17.8)	93 (26.5)		147 (23.0)	86 (18.0)		91 (48.1)	10 (4.8)	
No	596 (82.2)	258 (73.5)		492 (77.0)	392 (82.0)		98 (51.9)	200 (95.2)	

Data are expressed as *n* (%).

^a^ Median (Q1, Q3).

### 3.2 Correlation analysis between vitamin A status and RRTIs

The associations between vitamin A status and RRTIs were analyzed using multivariable logistic regression. As shown in Table 3, the ORs for VA insufficiency were 1.55 (95% CI: 1.30–1.84) and 2.30 (95% CI: 1.82–2.90) for VAD compared with the VA adequate group. Model 1 adjusted for age and sex, the ORs for VA insufficiency and deficiency were 1.56 (95% CI: 1.31–1.86), 2.31 (95% CI: 1.82–2.93); model 2 was adjusted for BMI z-scores, fever, infectious factors and VA supplementation on the basis of model 1, with an OR of 1.32 (95% CI: 1.09–1.60) for VA insufficiency and 1.95 (95% CI: 1.50–2.55) for VAD.

**Table 3:** Associations between vitamin A status and RRTI in multivariable logistic regression models.

Characteristics	Vitamin A sufficiency	Vitamin A insufficiency	Vitamin A deficiency	*P*_trend_
Crude OR (95% CI)	Ref	1.55 (1.30–1.84)	2.30 (1.82–2.90)	<0.001
Model 1 OR (95% CI)	Ref	1.56 (1.31–1.86)	2.31 (1.82–2.93)	<0.001
Model 2 OR (95% CI)	Ref	1.32 (1.09–1.60)	1.95 (1.50–2.55)	<0.001

Model 1, adjusted for age and sex; Model 2, adjusted for age, sex, BMI *z*-scores, fever, symptoms of respiratory infection and vitamin A preparation.

The associations were also analyzed according to current RTI symptoms. In children with current RTI symptoms, the ORs for the serum VA insufficiency and deficiency groups compared with the sufficient group were 1.33 (95% Cl: 1.05–1.69) and 4.74 (95% Cl: 3.19–7.03), respectively; model 1 adjusted for sex and age, the ORs for the insufficiency and deficiency groups were 1.36 (95% Cl: 1.06–1.73) and 5.25 (95% Cl: 3.51–7.87); model 2 had ORs of 1.48 (95% Cl: 1.13–1.94) and 6.51 (95% CI: 4.18–10.14). In children without symptoms of respiratory infection, the ORs at model 2 were 1.63 (95% Cl: 1.21–2.19) and 1.57 (95% Cl: 1.02–2.43) for the groups with VA insufficiency and deficiency, respectively, compared to that with VA sufficiency (Table 4).

**Table 4:** Associated factors screened in multivariable logistic regression models among children with or without current RTI symptoms.

Characteristics	Vitamin A sufficiency	Vitamin A insufficiency	Vitamin A deficiency	*P*_trend_
Current symptoms of respiratory infection				
Crude OR (95% CI)	Ref	1.33 (1.05–1.69)	4.74 (3.19–7.03)	<0.001
Model 1 OR (95% CI)	Ref	1.36 (1.06–1.73)	5.25 (3.51–7.87)	<0.001
Model 2 OR (95% CI)	Ref	1.48 (1.13–1.94)	6.51 (4.18–10.14)	<0.001
No symptoms of respiratory infection				
Crude OR (95% CI)	Ref	1.46 (1.10–1.94)	1.18 (0.80–1.74)	0.031
Model 1 OR (95% CI)	Ref	1.50 (1.13–2.01)	1.19 (0.80–1.77)	0.022
Model 2 OR (95% CI)	Ref	1.63 (1.21–2.19)	1.57 (1.02–2.43)	0.004

Model 1, adjusted for age and sex; Model 2, adjusted for age, sex, BMI *z*-scores, fever and vitamin A preparation.

### 3.3 Correlation analysis between dietary intake and RRTIs

**Figure 1:**
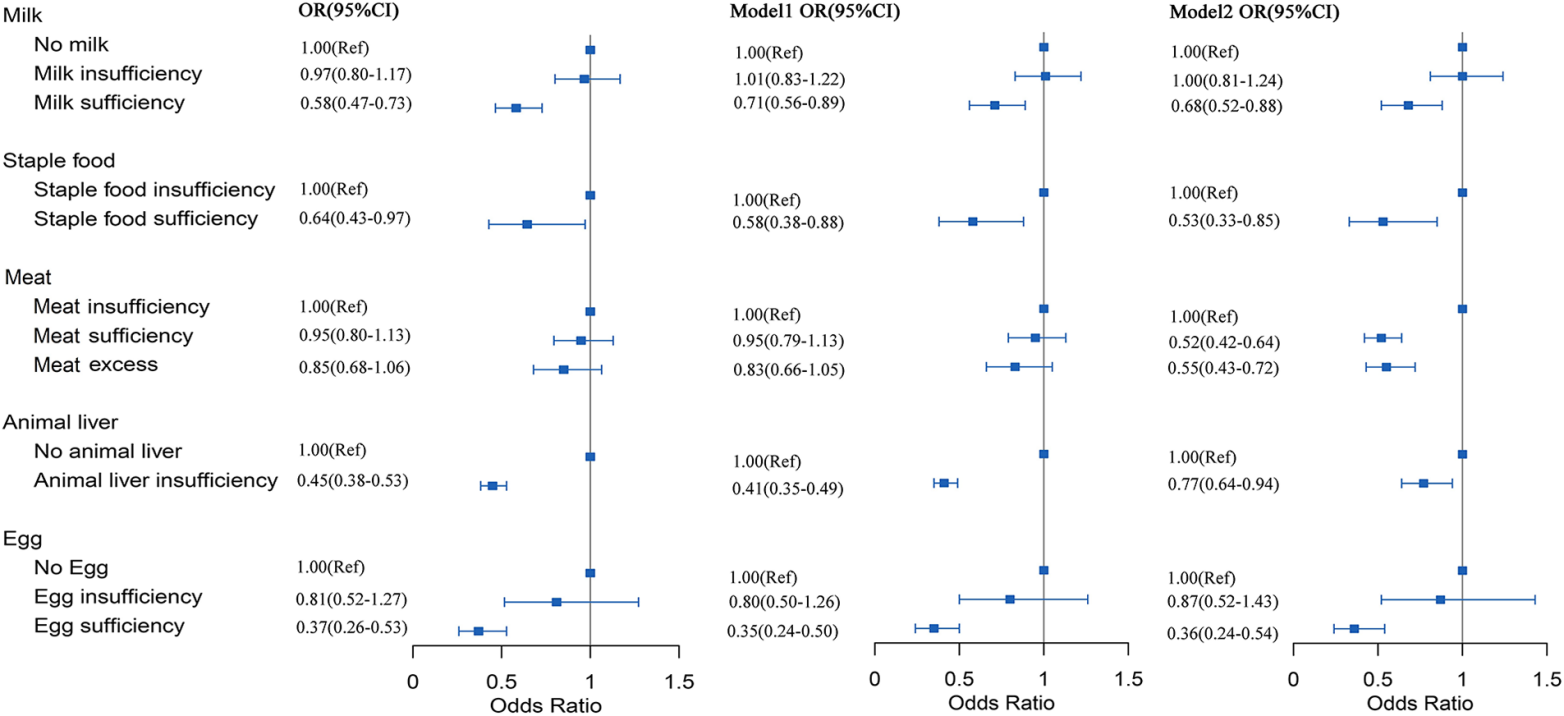
Correlation between intake of vitamin A-rich foods and RRTIs. Point estimates were the ORs and horizontal lines represented the 95% CI. OR, odds ratio; CI, confidence intervals. Model 1, adjusted for age (quantile) and sex; Model 2, adjusted for age, sex, BMI z-scores, fever and symptoms of respiratory infection.

Logistic regression models were used to determine whether lower dietary intake was associated with RRTIs. The OR for sufficient milk intake compared to no milk intake was 0.68 (95% CI: 0.52–0.88); the OR for adequate staple intake compared to insufficient intake was 0.53 (95% CI: 0.33–0.85); the OR for sufficient meat intake compared to insufficient meat intake was 0.52 (95% CI: 0.42–0.64); no children had adequate animal liver intake; compared to no animal liver intake, the OR for insufficient intake of animal liver was 0.77 (95% CI: 0.64–0.94); the OR for adequate intake of eggs was 0.36 (95% CI: 0.24–0.54) compared to no intake in model 2 (Figure 1).

The frequency of intake of 17 food items was obtained from 2,053 children aged 2–14 years old according to the recommended intake for that age. Through PCA, the positive factor loadings more than 0.60 were included in each dietary pattern and the dietary patterns were named according to the dietary components of the dominant food groups. The fruit and vegetable pattern included: vegetables (carrots, kale borecole, spinach, broccoli, pumpkin, Chinese cabbage), fruits (apple, pear, banana, apricot, watermelon, orange) and soybean; staples, meat and egg pattern included: staples (cereals and potatoes), meat and egg; and the third dairy pattern included dairy products (Table 5). Children were divided into quartiles according to the factor scores of each dietary pattern. The relationship between dietary intake and the risk of developing RRTIs was analyzed by logistic regression. For the vegetable-fruit model, the OR of Q2 and Q3 decreased with increasing scores compared to Q1; while the ORs of Q4 were not statistically significant. For the staples-meat-egg model, the OR decreased with higher factor scores, and in model 3 the Q4 OR was 0.15 (95% CI: 0.09–0.25). In the milk model, the OR for Q2 and Q3 were not statistically significant compared to Q1 and were <1 at Q4 regardless of what factors were balanced, with an OR of 0.60 (95% CI: 0.45–0.80) (Table 6).

**Table 5:** Rotated factor loading matrix of dietary patterns from PCA among children of 2–14 years.

	Vegetable-fruit	Staples-meat-egg	Milk
Carrots	0.89	–	–
Kale borecole	0.91	–	–
Spinach	0.93	–	–
Broccoli	0.87	–	–
Pumpkin	0.92	–	–
Chinese cabbage	0.94	–	–
Apple	0.84	–	–
Pear	0.93	–	–
Banana	0.91	–	–
Apricot	0.92	–	–
Watermelon	0.94	–	–
Orange	0.92	–	–
Soybean	0.79	–	–
Staples	–	0.88	–
Meat	–	0.62	–
Egg	–	0.84	
Milk	–	–	0.99

Based on principal component analysis. Factor loading ≥0.60 was included in each factor.

**Table 6:** Multiple logistic regression for the risk of RRTI according to the quartiles of dietary pattern scores (*n* = 2,053).

	Model	Q2	Q3	Q4	*P*_trend_
Vegetable-fruit	Model 1	0.55 (0.43–0.71)	0.41 (0.32–0.53)	1.11 (0.87–1.42)	<0.001
	Model 2	0.55 (0.43–0.72)	0.42 (0.32–0.55)	1.11 (0.86–1.42)	<0.001
	Model 3	0.94 (0.70–1.25)	0.74 (0.55–1.00)	1.48 (1.12–1.95)	<0.001
Staples-meat-egg	Model 1	1.09 (0.86–1.40)	0.80 (0.63–1.02)	0.50 (0.39–0.65)	<0.001
	Model 2	0.81 (0.60–1.11)	0.46 (0.32–0.67)	0.22 (0.14–0.35)	<0.001
	Model 3	0.75 (0.53–1.05)	0.34 (0.23–0.52)	0.15 (0.09–0.25)	<0.001
Milk	Model 1	0.85 (0.66–1.08)	0.84 (0.66–1.07)	0.54 (0.42–0.69)	<0.001
	Model 2	0.87 (0.68–1.11)	0.83 (0.65–1.07)	0.55 (0.43–0.72)	<0.001
	Model 3	0.97 (0.74–1.27)	0.81 (0.62–1.07)	0.60(0.45–0.80)	0.001

Model 1, unadjusted; Model 2, adjusted for age and sex; Model 3, adjusted for age, sex, BMI *z*-scores, fever and symptoms of respiratory infection.

## 4 Discussion

RRTIs are common in children and severe RRTIs may be harmful to both the physical and mental health of children (4). In addition to pathological damage to the upper and lower respiratory tracts, recurrent episodes of RRTIs may have adverse effects on neighboring organs and lead to otitis media in children; secondary immune responses can lead to acute glomerulonephritis and myocarditis, resulting in a poor prognosis for children with RRTIs and an increase in the financial burden of treatment (25, 26). The pathogenesis of RRTIs is complex and multifactorial. Previous studies have shown that the disruption of the barrier function of the respiratory tract and impaired immune function may be closely linked with RRTIs. The association between VAD and development of RRTI in children has been found recently (21). VA is a very important fat-soluble vitamin to human and plays an immunomodulatory role in the human body, especially in the maintenance of airway epithelial cell integrity and immune function (27, 28). Studies have shown that approximately 140–250 million children under 5 years of age worldwide have VAD (29). Children with chronic VAD are susceptible to a variety of diseases, e.g., RRTIs, growth restriction and immune disorders (30, 31). In this study, our data showed that (1) serum VA insufficiency or deficiency was positively associated with the incidence of RRTIs; (2) serum VA insufficiency or deficiency in children with respiratory infection symptoms was positively associated with the occurrence of RRTIs; (3) daily dietary intakes of vitamin A were also associated with the occurrence of RRTIs.

It has been found that the incidence of respiratory infections in children with VAD is about two times higher than in normal children (32), and that VAD children with normal growth and development have higher prevalence of respiratory infections than non-VAD peers with delayed growth (33, 34). In addition, depending on the definition of RRTIs, a child diagnosed with RRTIs may either be in the midst of a recurrent and ongoing period of infection or may have been free of reinfection for some time and be in the process of getting better. Therefore, the definition of RRTIs based on the number of infections alone may not be able to distinguish between these two conditions, so in this study, the presence or absence of respiratory infection symptoms was collected from children at the time of enrollment, and the risk of RRTIs was further confirmed by statistical methods such as stratified analysis and logistic regression for children with respiratory infection symptoms and lower serum VA levels.

VA for human is derived from both animal (retinoids) and plant (provitamin A carotenoids) sources (33). Animal food sources, e.g., milk, eggs and meats were analyzed in this study. Some findings have suggested that the absorption of fat-soluble vitamins can be enhanced in presence of dietary fat, with dairy products playing an important role in children's diets (35–37). We found that a higher intake of milk, meat, animal livers and eggs was associated with a lower risk of RRTIs. Although no children in this study consumed sufficient animal livers, consumption of animal livers somewhat played a protective role in decreasing the risk of RRTIs compared with no intake of animal livers. In addition, we found that all the three dietary patterns were protective against developing RRTIs. Among the vegetable-fruit models, Q2 and Q3 were associated with a reduced risk of RRTIs; higher adherence to the staples-meat-egg model and the milk model was associated with a lower risk of RRTIs. Therefore, the risk of developing RRTIs was reduced when VA-rich foods were sufficiently consumed; adequate intake of VA-rich foods in children was vital to reducing the risk of developing RRTIs.

The relation between compliance with dietary recommendations and development of upper respiratory tract infections in children has been studied (38). This study was the first to examine the relation between dietary intake and patterns and RRTIs. The association of the intake of high protein and VA-rich animal foods (milk, eggs, animal liver) with RRTIs was mainly investigated. The analysis on the relation between the frequency of intake of VA-rich plant food intake, other than portions, and RRTIs was conducted. Therefore, future study would aim at investigating the intake and specific VA content of multiple food groups and exploring their relation with RRTIs to provide strong evidence for appropriate VA intake in children's diet to prevent RRTIs.

The current study has some limitations. First, parents or guardians were asked about food allergies in their children only during sample collection, but the bias in reporting RRTIs might exist with the assessment of current RTI symptoms. Second, the diagnostic criteria of pediatric RRTIs were based on the frequencies of upper and lower respiratory tract infections according to children's age groups, resulting in possible recall bias (or other types of bias). Similarly, other potential confounding factors should be considered. Third, this was a case-control study and thus prospective research was warranted in the future. More future investigation would focus on the types and specific intakes of vitamin A-rich foods as well as vitamin A supplements, and further elucidation of the molecular mechanisms of specific fat-soluble vitamin deficiency leading to RRTIs.

## 5 Conclusions

In summary, low serum vitamin A concentrations were associated with RRTIs in children in Northeast China; lower intake of vitamin A-rich foods was also associated with higher risk of development of RRTIs. Therefore, for children with RRTIs or those vulnerable to RRTIs in Northeast China, it was crucial to consume adequate vitamin A-rich foods or supplements regularly, if necessary, to maintain normal serum vitamin A levels for a lower risk of RRTIs.

## Data Availability

The datasets presented in this article are not readily available because the original contributions presented in this study are included in the article; further inquiries can be directed to the corresponding author. Requests to access the datasets should be directed to Yan Zhao, amyzhaosb@163.com.

## References

[1] Zhang J Sun RR Yan ZX Yi WX Yue B. Correlation of serum vitamin A, D, and E with recurrent respiratory infection in children. Eur Rev Med Pharmacol Sci. (2019) 23(18):8133–8. 10.26355/eurrev_201909_19033 31599442

[2] Yin J Xu B Zeng X Shen K. Broncho-Vaxom in pediatric recurrent respiratory tract infections: a systematic review and meta-analysis. Int Immunopharmacol. (2018) 54:198–209. 10.1016/j.intimp.2017.10.032 29154122

[3] Imdad A Mayo-Wilson E Haykal MR Regan A Sidhu J Smith A Vitamin A supplementation for preventing morbidity and mortality in children from six months to five years of age. Cochrane Database Syst Rev. (2022) 3(3):CD008524. 10.1002/14651858.CD008524.pub4 35294044 PMC8925277

[4] Horton S Blum LS Diouf M Ndiaye B Ndoye F Niang K Delivering vitamin A supplements to children aged 6–59 months: comparing delivery through campaigns and through routine health services in Senegal. Curr Dev Nutr. (2018) 2(4):nzy006. 10.1093/cdn/nzy006 30019030 PMC6041955

[5] Hollm-Delgado MG Piel FB Weiss DJ Howes RE Stuart EA Hay SI Vitamin A supplements, routine immunization, and the subsequent risk of plasmodium infection among children under 5 years in Sub-Saharan Africa. Elife. (2015) 4:e03925. 10.7554/eLife.03925 25647726 PMC4383226

[6] Dong F Yu H Ma J Wu L Liu T Lv G Exploring association between gastrointestinal heat retention syndrome and recurrent respiratory tract infections in children: a prospective cohort study. BMC Complement Altern Med. (2016) 16:82. 10.1186/s12906-016-1062-8 26921252 PMC4769561

[7] Li H Cui QK Li Z Li J Li F. Clinical observation of the effect of modified Ginseng-Schisandra decoction (MGSD) on trace elements and immune function in children with spleen deficiency syndrome after recurrent respiratory tract infection (RRTI): a randomized controlled trial. Transl Pediatr. (2021) 10(6):1692–700. 10.21037/tp-21-243 34295784 PMC8261592

[8] Schatorjé EJ de Jong E van Hout RW García Vivas Y de Vries E. The challenge of immunoglobulin-G subclass deficiency and specific polysaccharide antibody deficiency–a Dutch pediatric cohort study. J Clin Immunol. (2016) 36(2):141–8. 10.1007/s10875-016-0236-y 26846287

[9] Pasternak G Lewandowicz-Uszyńska A Pentoś K. Disorders of humoral immunity in children with IgG subclass deficiency and recurrent respiratory infections. Adv Exp Med Biol. (2018) 1108:99–106. 10.1007/5584_2018_263 30182338

[10] Zhou B Niu W Liu F Yuan Y Wang K Zhang J Risk factors for recurrent respiratory tract infection in preschool-aged children. Pediatr Res. (2021) 90(1):223–31. 10.1038/s41390-020-01233-4 33173178

[11] de Benedictis FM Bush A. Recurrent lower respiratory tract infections in children. Br Med J. (2018) 362:k2698. 10.1136/bmj.k2698 30002015

[12] Debelo H Novotny JA Ferruzzi MG. Vitamin A. Adv Nutr. (2017) 8(6):992–4. 10.3945/an.116.014720 29141980 PMC5683001

[13] Timoneda J Rodríguez-Fernández L Zaragozá R Marín MP Cabezuelo MT Torres L Vitamin A deficiency and the lung. Nutrients. (2018) 10(9):1132. 10.3390/nu10091132 30134568 PMC6164133

[14] Stone CA Jr McEvoy CT Aschner JL Kirk A Rosas-Salazar C Cook-Mills JM Update on vitamin E and its potential role in preventing or treating bronchopulmonary dysplasia. Neonatology. (2018) 113(4):366–78. 10.1159/000487388 29514147 PMC5980725

[15] Tang K Eilerts H Imohe A Adams KP Sandalinas F Moloney G Evaluating equity dimensions of infant and child vitamin A supplementation programmes using demographic and health surveys from 49 countries. BMJ Open. (2023) 13(3):e062387. 10.1136/bmjopen-2022-062387 36918231 PMC10016247

[16] Brown CC Noelle RJ. Seeing through the dark: new insights into the immune regulatory functions of vitamin A. Eur J Immunol. (2015) 45(5):1287–95. 10.1002/eji.201344398 25808452 PMC4426035

[17] Hall JA Grainger JR Spencer SP Belkaid Y. The role of retinoic acid in tolerance and immunity. Immunity. (2011) 35(1):13–22. 10.1016/j.immuni.2011.07.002 21777796 PMC3418663

[18] Salo PM Mendy A Wilkerson J Molsberry SA Feinstein L London SJ Serum antioxidant vitamins and respiratory morbidity and mortality: a pooled analysis. Respir Res. (2022) 23(1):150. 10.1186/s12931-022-02059-w 35681205 PMC9178544

[19] Vlieg-Boerstra B de Jong N Meyer R Agostoni C De Cosmi V Grimshaw K Nutrient supplementation for prevention of viral respiratory tract infections in healthy subjects: a systematic review and meta-analysis. Allergy. (2022) 77(5):1373–88. 10.1111/all.15136 34626488

[20] Wang X Li X Jin C Bai X Qi X Wang J Association between Serum vitamin A levels and recurrent respiratory tract infections in children. Front Pediatr. (2021) 9:756217. 10.3389/fped.2021.756217 35004539 PMC8740126

[21] Abdelkader A Wahba AA El-Tonsy M Zewail AA Shams Eldin M. Recurrent respiratory infections and vitamin A levels: a link? It is cross-sectional. Medicine (Baltimore). (2022) 101(33):e30108. 10.1097/MD.0000000000030108 35984160 PMC9388016

[22] Zhang X Ding F Li H Zhao W Jing H Yan Y Low serum levels of vitamins A, D, and E are associated with recurrent respiratory tract infections in children living in northern China: a case control study. PLoS One. (2016) 11(12):e0167689. 10.1371/journal.pone.0167689 27936124 PMC5147939

[23] Guidelines for the treatment of pediatric recurrent respiratory tract infections in children. Zhongguo Shi Yong Er Ke Za Zhi. (2022) 37(3):161–68.

[24] Chinese dietary guidelines. Chinese nutrition society. Available at: http://dg.en.cnsoc.org.

[25] Khan Laghari I Nawaz T Mustafa S Jamali AA Fatima S. Role of multi-strain probiotics in preventing severity and frequency of recurrent respiratory tract infections in children. BMC Pediatr. (2023) 23(1):505. 10.1186/s12887-023-04338-x 37817096 PMC10566059

[26] Braido F Melioli G Nicolini G Canonica GW. Prevention of recurrent respiratory tract infections: a literature review of the activity of the bacterial lysate lantigen B. Eur Rev Med Pharmacol Sci. (2023) 27(16):7756–67. 10.26355/eurrev_202308_33430 37667954

[27] Niu H Wang R Jia YT Cai Y. Pidotimod, an immunostimulant in pediatric recurrent respiratory tract infections: a meta-analysis of randomized controlled trials. Int Immunopharmacol. (2019) 67:35–45. 10.1016/j.intimp.2018.11.043 30530167

[28] Chen G Weiskirchen S Weiskirchen R. Vitamin A: too good to be bad? Front Pharmacol. (2023) 14:1186336. 10.3389/fphar.2023.1186336 37284305 PMC10239981

[29] Feleszko W Marengo R Vieira AS Ratajczak K Mayorga Butrón JL. Immunity-targeted approaches to the management of chronic and recurrent upper respiratory tract disorders in children. Clin Otolaryngol. (2019) 44(4):502–10. 10.1111/coa.13335 30920131 PMC6850198

[30] Cribb VL Northstone K Hopkins D Emmett PM. Sources of vitamin A in the diets of pre-school children in the avon longitudinal study of parents and children (ALSPAC). Nutrients. (2013) 5(5):1609–21. 10.3390/nu5051609 23676550 PMC3708340

[31] Martini S Rizzello A Corsini I Romanin B Fiorentino M Grandi S Vitamin A deficiency due to selective eating as a cause of blindness in a high-income setting. Pediatrics. (2018) 141(Suppl 5):S439–44. 10.1542/peds.2016-2628 29610168

[32] Raposo SE Fondell E Ström P Bälter O Bonn SE Nyrén O Intake of vitamin C, vitamin E, selenium, zinc and polyunsaturated fatty acids and upper respiratory tract infection-a prospective cohort study. Eur J Clin Nutr. (2017) 71(4):450–7. 10.1038/ejcn.2016.261 28074891

[33] Hawke K King D van Driel ML McGuire TM. Homeopathic medicinal products for preventing and treating acute respiratory tract infections in children. Cochrane Database Syst Rev. (2022) 12(12):CD005974. 10.1002/14651858.CD005974.pub6 36511520 PMC9746041

[34] Guo M Zhu J Yang T Lai X Lei Y Chen J Vitamin A and vitamin D deficiencies exacerbate symptoms in children with autism spectrum disorders. Nutr Neurosci. (2019) 22(9):637–47. 10.1080/1028415X.2017.1423268 29338670

[35] Yang B Huang S Yang N Cao A Zhao L Zhang J Porcine bile acids promote the utilization of fat and vitamin A under low-fat diets. Front Nutr. (2022) 9:1005195. 10.3389/fnut.2022.1005195 36245518 PMC9554479

[36] Unlu NZ Bohn T Clinton SK Schwartz SJ. Carotenoid absorption from salad and salsa by humans is enhanced by the addition of avocado or avocado oil. J Nutr. (2005) 135(3):431–6. 10.1093/jn/135.3.431 15735074

[37] Zhang Y Du Z Ma W Chang K Zheng C. Vitamin A status and recurrent respiratory infection among Chinese children: a nationally representative survey. Asia Pac J Clin Nutr. (2020) 29(3):566–76. 10.6133/apjcn.202009_29(3).0016 32990617

[38] van der Gaag E Brandsema R Nobbenhuis R van der Palen J Hummel T. Influence of dietary advice including green vegetables, beef, and whole dairy products on recurrent upper respiratory tract infections in children: a randomized controlled trial. Nutrients. (2020) 12(1):272. 10.3390/nu12010272 31968697 PMC7019298

